# Construction Notes

**Published:** 1896-03-14

**Authors:** 


### CONSTRUCTION NOTES.

**Cottage Hospital at Porth, Rhondda Valleys.**

This hospital, overlooking the Rhondda Valleys, is an imposing structure, with handsome entrance and portico. Just inside a brass slab bears the inscription, "This tablet was erected to commemorate the generous gift to this institution of £800 by Mr. George Thomas, of Ystradmynach." The hospital buildings are of local stone, with red brick dressings, and contain accommodation for eight patients on the ground floor, and two wards on the first floor for private patients, for cases requiring isolation. The cost of the buildings is about £3,000, and it is satisfactory to note that they were opened free of debt. The whole of the work was carried out by Messrs. Charles Jenkins and Sons, contractors, of Porth.

**Cottage Hospital for Accrington District.**

An imposing demonstration accompanied the ceremony of laying the foundation-stone of this cottage hospital. It is to be built from plans prepared by Messrs. Haywood and Harrison, and recommended from a limited competition by Sir Douglas Galton. The building, "Renaissance" in style, will be of red plastic brick, with Yorkshire stone dressings, and will have a commanding frontage, towards the town, with a southern aspect. The hospital has been planned with the whole of the wards upon the ground floor, and arranged in such a manner as to get the greatest amount of sunshine. The entrance for accident patients is at the side, and in close proximity to the receiving and operating rooms, and entirely away from the sight of the in-patients. The male and female wards, containing sixteen and twenty-two beds, and two cots, are placed at the right and left respectively of a wide corridor, with a nurse's room on each side, placed between the large and small wards, with inspection windows in each. The matron's sitting-room commands the front entrance, and the board-room is also conveniently placed near the entrance. The operating theatre (with anæsthetic and receiving rooms) is in the rear, for the purpose of getting the north light; and the kitchen, scullery, &c., are as far away from the rest of the rooms as possible, and separated by ventilated lobbies and halls. On the ground floor are also the ward kitchen, dispensary stores, and splint rooms. On the first floor are the nurses' sitting and bed rooms, bath-rooms, &c., and accommodation for the servants. Special attention has been paid to ventilation and warming, and all the rooms are well lighted, ventilating sun-lights being employed in the larger wards. The floors of the wards, operating-room, and corridors are to be of long and grooved hard wood polished, and the basement concreted throughout. The building will be set back from the main road, and the surrounding land laid out with flower beds, and will prove a decided acquisition to the town, both ornamentally as well as for the purposes for which it is intended.

